# Editorial: Plasticity and metabolic switching in adipose tissue macrophages

**DOI:** 10.3389/fimmu.2023.1233791

**Published:** 2023-06-13

**Authors:** Jerry Zhang, Junji Xing

**Affiliations:** ^1^ Department of Surgery and Immunobiology and Transplant Science Center, Houston Methodist Research Institute, Houston Methodist, Houston, TX, United States; ^2^ Department of Cardiovascular Sciences, Houston Methodist Research Institute, Houston Methodist, Houston, TX, United States

**Keywords:** innate immunity, adipose tissue macrophages, plasticity, phenotype, metabolism, therapeutic target, obesity, metabolic diseases

Macrophages are frontier soldiers of innate immunity. Adipose tissue macrophages (ATMs), originally identified by the expression of the macrophage marker F4/80 in murine fat depots, are the most abundant immune cells in adipose tissue, representing more than half of leukocytes in depots from lean and obese animals (1). ATMs have an important role in maintaining adipose tissue homeostasis and contributing to the metabolically harmful chronic inflammation in obesity associated diseases (2). ATMs are a heterogenous population of cells with ‘hard wired’ diversity brought upon by distinct developmental lineages in the adipose tissue of lean and obese animals (3). ATMs exhibit phenotypic plasticity requiring polarization switching between pro- and anti-inflammatory phenotypes and functional diversity between innate and adaptive immunity in lean and obese mice and humans (4). ATMs also have complex functions in metabolism inflammation and important adaptive functions in lipid homeostasis in obesity-related diseases (2). Although research on ATMs is accumulating, there is still much to uncover regarding the developmental origin, phenotypic plasticity, functional diversity, and metabolism regulation of ATMs in obesity-related diseases.

This Research Topic “*Plasticity and Metabolic Switching in Adipose Tissue Macrophages*” highlights 10 recent studies that investigate the metabolic regulation, phenotype, developmental origin, and polarization regulation of ATMs, and summarize the plasticity, regulatory mechanisms, and therapeutic targets of ATMs in obesity-related diseases.

The study of the mechanisms underlying the conversion from the metabolically healthy obese (MHO) to the metabolically unhealthy obese (MUO) represents a substantial opportunity for the development of personalized stratified risk therapies in obesity-related diseases (5). Johnson et al. investigated the mechanisms underlying the transition from MHO to MUO and found a novel subset of CD95+ proinflammatory macrophages may mediate the switch from MHC to MUC. The authors showed the MHO mice (ApoE^-/-^miR155^-/-^ mouse model) shift to MUO with increased vascular inflammation and atherosclerosis after extended (24 weeks) high-fat diet (HFD) feeding. Mechanistically, they found the CD95^+^CD86^-^ subset of proinflammatory ATMs were increased to activate aortic endothelial cells for promoting vascular inflammation in MUO mice.

The heterogeneity, phenotype, and developmental origin of ATMs remain unknown in obese individuals. Felix et al. analyzed white adipose tissue (WAT) during homeostasis and diet interventions using single-cell mass cytometry and genetic lineage tracking models. The authors found there were eight kinetically evolving CD206^+^ ATMs (defined by TIM4, CD163 and MHCII) and two CD206^-^ ATMs in WAT of lean mice. They showed TIM4^-^CD163^+^, TIM4^-^CD163^-^ and CD206^-^ ATMs were mainly bone marrow-derived, whereas the proliferating TIM4^+^CD163^+^ ATMs were of embryonic origin. Additionally, a HFD induced massive infiltration of CD206^-^ ATMs and selective down-regulation of MHC II on TIM4^+^ ATMs, suggesting that the development origin and environment jointly regulate the functional malleability of resident ATMs.

The underlying mechanisms of tissue remodeling, immunomodulation, and polarization of ATMs in obese patients with tumor are still elusive. Micallef et al. identified the C1q/TNF-related protein family member C1qtnf3 as one of the most regulated genes in tumor-associated inguinal adipose tissue from HFD-induced obese mice. They showed administration of C1QTNF3 neutralizing antibodies inhibited ATMs accumulation in tumor-associated inguinal adipose tissue while tumor growth was unaffected. The C1QTNF3 treatment promoted polarization of M2-type macrophages to M1-like macrophages through activation ERK and Akt pathway. These results suggest the immunomodulatory effects of C1qTNF3 in polarization of ATMs and adipose tissue remodeling. Thibaut et al. reported the relationship of cellular metabolism and macrophage polarization. They showed that disruption of cellular metabolism influenced cytokine secretion and expression of crucial inflammatory genes in M1 and M2 macrophages, highlighting the need for specific metabolic functions in regulating macrophage polarization. Pan et al. reviewed the potential regulatory mechanisms underlying ATMs polarization induced by autocrine and paracrine factors. A better understanding of how ATMs polarize may provide novel therapeutic strategies for obesity-related diseases.

ATMs infiltration into adipose tissue plays pathogenic role in inducing adipose tissue dysfunction and contributes to obesity-induced inflammation and metabolic diseases. Li et al. summarized the latest research on the heterogeneity of ATMs in adipose tissue and presented the identities of the newly discovered ATMs subtypes. They also discussed macrophage-targeting strategies to ameliorate obesity-related inflammation and metabolic diseases. Liang et al. summarized the factors affecting the polarization of ATMs and the pathogenic mechanisms of ATMs in metabolic diseases like obesity and diabetes. They also reviewed the progression of ATMs as a potential therapeutic target for treating obesity and diabetes. Matz et al. reviewed current knowledge on regulatory networks critical to plasticity and multifaceted response of ATMs in the complex adipose tissue microenvironment. This will give a clue on how to target ATMs to lessen obesity-associated health risks.

The above description shows the important roles of ATMs in obesity and metabolic disorders. However, recent studies have identified the unique role and regulation of ATMs in thermogenic adipose tissue to regulate energy expenditure and systemic energy homeostasis. Rahman and Jun summarized the current understanding of ATMs in thermogenic fat niches and the critical roles of four distinct subsets of ATMs in adaptive thermoregulation. Hepatic glucose production (HGP) is fine-regulated to maintain physiological concentration of blood glucose, whereas aberrant HGP leads to hyperglycemia in obesity-associated diabetes. Tao et al. reviewed several pathways by which ATMs remotely regulate HGP and summarized emerging therapeutic targets to treat metabolic disorders in morbid obesity or diabetes based on ATMs-HGP axis.

Finally, we would like to thank all the authors for entrusting us with their discoveries, and all the referees for their careful and insightful review. We believe that all the articles included in this Research Topic will be of interest to all researchers studying the role of ATMs in obesity-related diseases and will make them aware of how a clearer understanding of these mechanisms can guide future therapeutic treatments for obesity-related diseases.

## Author contributions

JZ and JX performed literature research and wrote the manuscript. All authors contributed to the manuscript and approved it for publication.
